# Recent Progress on Semiconductor-Interface Facing Clinical Biosensing

**DOI:** 10.3390/s21103467

**Published:** 2021-05-16

**Authors:** Mingrui Zhang, Mitchell Adkins, Zhe Wang

**Affiliations:** 1 School of Engineering, University of Manchester, Manchester M13 9PL, UK; mingrui.zhang-2@student.manchester.ac.uk; 2 Chemistry Department, Oakland University, Rochester, MI 48309, USA; mitchelladkins@oakland.edu

**Keywords:** biosensor, semiconductor, interface, nanomaterial, field-effect transistor

## Abstract

Semiconductor (SC)-based field-effect transistors (FETs) have been demonstrated as amazing enhancer gadgets due to their delicate interface towards surface adsorption. This leads to their application as sensors and biosensors. Additionally, the semiconductor material has enormous recognizable fixation extends, high affectability, high consistency for solid detecting, and the ability to coordinate with other microfluidic gatherings. This review focused on current progress on the semiconductor-interfaced FET biosensor through the fundamental interface structure of sensor design, including inorganic semiconductor/aqueous interface, photoelectrochemical interface, nano-optical interface, and metal-assisted interface. The works that also point to a further advancement for the trademark properties mentioned have been reviewed here. The emergence of research on the organic semiconductor interface, integrated biosensors with Complementary metal–oxide–semiconductor (CMOS)-compatible, metal-organic frameworks, has accelerated the practical application of biosensors. Through a solid request for research along with sensor application, it will have the option to move forward the innovative sensor with the extraordinary semiconductor interface structure.

## 1. Introduction

The larger part of current biosensors are classified by four distinctive standards. Refinement is a rule determined by affinity, catalytic, transmembrane, and cell sensors. However, the transmembrane and cell sensors are based on affinity or catalytic standards as well. Current investigation and improvement has made strides in sensitivity, selectivity, and stability of biosensors. In particular, there is an expanding drift towards scaling down those spectrometers, chromatographs, and detectors, which are customarily utilized in bioanalytical chemistry, by applying smaller scale- and nano-fabrication procedures. The current biosensors are equipped with miniaturized instruments for clinical application which can be progressively hard to use and, in certain cases, this poses a major challenge, especially, in the study of optical and electroanalytical biosensors. The clinical application of any sensor requires the utilization of total analytic frameworks and consequently integration with t sample handling, separation, and even sample conditioning. For designing biosensors, the analysis takes place by coordinate conversion of biochemical information to electronic signal through appropriate transducers and interface. There is a common bottleneck within the advancement of biosensors facing clinical applications that is deficient stability, reproducibility, and sensitivity of their interface properties within the complex clinical samples. Designing the interfaces is the key for next-generation biosensors. Due to a large amount of electrical and optical transducers accessible in thin-film innovation, most improvements of innovative biosensors aim at the arrangement of a controlled interface structure in which the biomolecular units were organized and packed in a reproducible and controlled manner [1].

Semiconductor-based field-effect transistors (FETs) have attracted critical consideration because of their high delicate interface with fluids, which makes them viable as biosensors [2]. A semiconductor additionally has a few qualities, for example, warm affectability, photosensitivity, negative resistivity temperature, rectifiable, etc. In this manner, semiconductor materials can be utilized for control gadgets, optoelectronic gadgets, pressure sensors, thermoelectric refrigeration, and different applications other than assembling enormous scale coordinated circuits. There are numerous orders for sensors, yet there are two usually utilized groupings: one is arranged by the deliberate physical material tested, the other is characterized by the working rule of sensors [3]. Sensors arranged by estimated physical amounts are usually temperature sensor, moistness sensor, pressure sensor, uprooting sensor, stream sensor, fluid level sensor, power sensor, speeding up sensor, torque sensor, and so on [4,5,6,7,8,9,10,11,12,13,14,15,16].

Nanobiosensors dependent on semiconductor materials are appropriate for wearable biosensor applications on account of the speedy reaction time, enormous perceivable fixation run, high affectability, high consistency for solid detecting, and the ability to coordinate with other microfluidic and electronic useful groups. In particular, one-dimensional (1D) nanomaterials have huge surface-to-volume proportions and tunable physical properties [17,18]. These qualities can be utilized to accomplish better execution over mass materials in an assortment of utilizations, including hardware, optics and photonics, vitality stockpiling and change gadgets, natural and concoction sensors, intracellular conveyance of bioactive atoms, and restorative gadgets. To date, another class of natural semiconductor gadgets functionalized by the quick ionic movement in the room temperature liquid salts has been identified. This new sort of strong to-fluid interface is framed between natural semi-conductor single crystals and ionic fluids so that the structures fill in as quick exchanging organic field-effect transistors (OFETs) with the most noteworthy transconductance gm = ID/VG per square channel sheet, i.e., the most proficient reaction of the yield channel current ID to the input gate voltage VG among the OFETs at any point. OFETs are possible for cutting edge minimal effort de-indecencies that can be created by straightforward manufacturing forms [19]. Be that as it may, performance is constrained by the poor authority over the quantities of utilitarian nanowires present in gadgets. Top-down systems experience the ill effects of significant gear and use costs. In recent years, the interest for profoundly touchy and reusable biosensors has expanded in light of the fact that the applications dependent on biosensors have evolved gotten different, with the range including medicinal, rural, modern, and ecological fields [20].

**Scheme 1:**
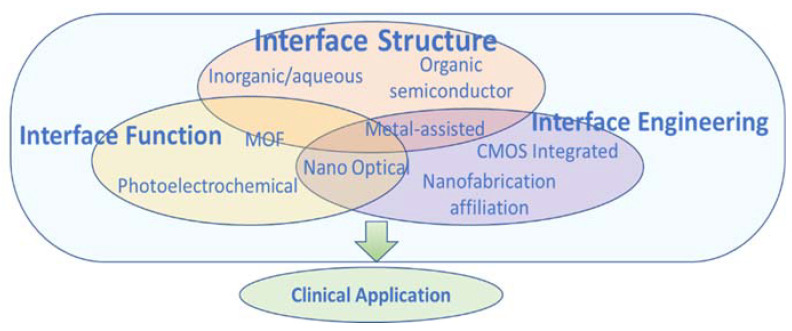
Summary framework of interface structure for sensor development.

Many review papers summarized a concessive overview on the recent achievements regarding the implementation of a metal oxide semiconductor (MOS) in biosensors used in biological and environmental systems [21,22,23,24,25,26,27,28,29,30,31,32,33,34,35,36,37,38,39,40,41,42,43,44,45,46,47,48,49]. So far there have been few works on the point of view of the unique interface semiconductor formed to oversee its application in the biosensor. The properties of FET sensors have been dramatically elevated by using the new sensor structure design and novel surface engineering enabled the sensor functions. In this review, we summarized the current progress on the semiconductor-based interface for the biosensor facing the application under the clinical environment. Besides traditional semiconductor/aqueous interfaces, the designs with new materials and fabrications including metal-assisted sensing material, membrane-less interface, and ionic liquid-based biosensor designs have been noted here (Scheme 1).

## 2. Current Progress Semiconductor Interfaced-Based Field-Effect Transistor (FET) Biosensors

### 2.1. Inorganic Semiconductor/Aqueous Interface

The many pathways of enhancing the sensing characteristics of metal oxides and metal sulfide by optimizing various parameters, such as synthesis methods, morphology, composition, and structure, have been explored in the last 10 years. For example, TiO_2_, ZnO, SnO_2_, and WO_3_, has been presented based on several aspects: synthesis method, morphology, functionalizing molecules, detection target, and limit of detection (LOD) [50,51,52]. Various developments have been made regarding applications of a metal oxide semiconductor (MOS)-based biosensors for environmental and biological systems. The recent developments have been summarized by the Enesca group in a concessive overview of the applications of metal oxides [53]. This describes the optimization of certain parameters for enhancing the sensing property of MOS. The parameters discussed include the method of synthesis, composition, structure, and morphology. Based on the aspects such as optimization of synthesis protocols, functionalizing agents, morphology, target detection, and limit of detection (LOD), the new representative metal oxides including TiO_2_, MoS_2_, graphene, and WSe_2_ with novel engineered methods are presented in this work. For the interface characterization, X-ray photoelectron spectroscopy (XPS) and ultraviolet photoelectron spectroscopy (UPS) have been widely used for investigating a wider range of organic–organic and metal–organic semiconductor interfaces [53]. Hill et al. [54], reported the use of UPS for the determination of binding energies associated with vacuum level position and highest occupied molecular orbital. Furthermore, they used UPS confirmation of possible chemical interaction that occurred at heterointerfaces. They found that most organic–organic interfaces exhibited the aligned vacuum level (with some exceptions), while all metal–organic interfaces exhibited strong interface dipoles which were responsible for an abrupt offset of the vacuum level. Moreover, the presence of dipoles in the metal–organic semiconductor interfaces where the Fermi level was completely unpinned within the gaps of semiconductors. This suggested that dipoles were not dependent on emptying or populating Fermi level-pinning gap states [54].

Here we summarize recent progress in this field with different engineering and material aspects.

#### 2.1.1. Nanofabrication-Affiliated FET

For the biosensor application, other than blood, other organic liquids are employed, for example, sweat, tears, and spit likewise contain gigantic biochemical analytes that can give significant data and are all the more promptly available contrasted with blood [55,56]. For PET-based biosensors, the entryway anode just needs to supply stable door inclination to the gadgets, which can be accomplished by an on-chip metal side cathode. For example, a profoundly conformal In_2_O_3_ nanoribbon FET biosensor with a completely coordinated on-chip gold side door, which has been overlaid onto different surfaces, was recently exhibited [57,58,59]. They have added side entryway examples to the source/channel shadow cover and have utilized a 5 μm ultra-flexible PET substrate and the subsequent shadow veil was then overlaid onto the PET substrate for the accompanying metal statement. Moreover, the chitosan ink was imprinted on PET In_2_O_3_ and the source and channel cushions utilizing an inkjet printing procedure. Also, the gold side door was utilized rather than the Ag/AgCl anode which expands the means and difficulty creating this. For affirming the detecting capacity of the biosensor the creators test the ionic affectability of the biosensor chip with a gold side or an Ag/AgCl cathode by PH detecting test [60].

(1) $$D-glucose+O_{2}\overset{glucose\;oxidase}{\to }gluconic\;acid+H_{2}O_{2}$$

(2) $$H_{2}O_{2}\to O_{2}+2H^{+}+2e^{-}$$

##### Nanostructured Arrays and Hierarchical Structure

In contrast to more traditional lithographical fabrication techniques, the template-based monolayer colloid crystal synthesis was considered as the promising alternative approach to the synthesis of microstructured and nanostructured arrays with varying morphology [61,62,63]. Using these approaches, researchers have been able to develop 2D ordered arrays including nanoring, nanoparticle, and pore arrays. In some cases, 1D nanostructures composed of the ordered nanorod, nanowire, and nanopillar arrays have also been developed. By reviewing recent progress in this field, synthesis strategies and morphology-related properties were presented as the association with these micro/nanostructured arrays, including their wettability, optical properties, photonic bandgap, and surface-enhanced Raman scattering [64].

The Johnson group report on the creation of a huge region In_2_O_3_ nanoribbon exhibits improved sensor properties, yet without utilizing bosses produced by means of electron beam lithography (EBL) [65]. The gathering utilized high-definition digital versatile discs (HD-DVD) for chemical lift-off lithography plasma-activated polydimethylsiloxane (CLL PDMS) stamps. These stamps are reusable and can be utilized for rehashed, wafer-scale CLL designing. Atomic Force Micrographs of designed PDMS surfaces affirmed exact replication of HD-DVD highlights with profundities of ~60 nm. Scanning Electron Microscopy (SEM) of CLL-designed self-assembled monolayer (SAMs) affirmed the nearness of 1D highlights (200 nm linewidths) over huge zones, coordinating those on the stamps. The widths (~200 nm) and statures (~3 nm) of the metal oxide nanoribbons are the smallest, as far as anyone is concerned, and created by means of top-down approaches. To assess the exhibition of In_2_O_3_ in gadgets, FETs were built in a base door, top-contact arrangement. Three thousand to 200-nm-wide In_2_O_3_ nanoribbon were consolidated into every FET device. This exhibited superior FETs created utilizing In_2_O_3_ nanoribbons having transporter mobilities up to 13.7 cm^2^ V^−1^ s^−1^ and on/off current proportions >10. These nanoribbon FETs have comparable electronic properties to In_2_O_3_ slim film transistors, however, they have higher surface-to-volume proportions. The presentation of these gadgets, for example, current on/off proportion, surpassed those recently detailed for 1D nanowire-based gadgets manufactured by means of a base-up approach. The nanostructures, for example, In_2_O_3_ nanoribbons and others, will be helpful in nanoelectronics and biosensors. The method shown here will empower these applications and require minimal effort, with huge zone designing methodologies to empower an assortment of materials and structure geometries in nanoelectronics [31].

**Figure 1:**
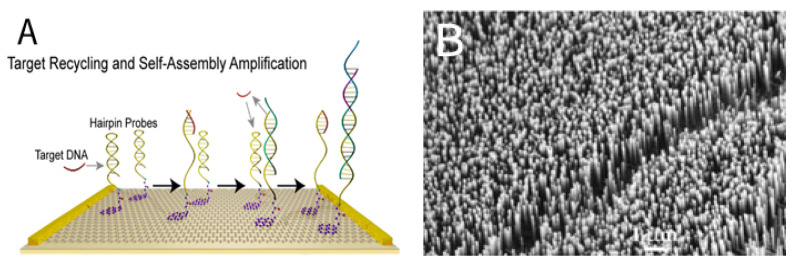
(**A**) Target recycling and self-assembly amplification. Reprinted with permission from ref. [65]. 2018 American Chemistry Society (**B**) The epitaxial growth of ZnO nanowires on the plane of a (110) sapphire substrate can be readily seen by examination of the crystal structures of ZnO and sapphire). Reprinted with permission from ref. [66]. 2002 Wiley-VCH.

Yang et al. [66] have discussed physical properties, rational growth, and integration of nanowires using the previously reported results from their laboratory. They proposed that vapor–solid-liquid (VLS) crystal growth mechanism can be used for controlling the growth position, diameter, composition, and orientation of nanowires. These nanowires were also investigated for their thermal stability and optical characteristic. They conclude that ZnO nanowires having defined surfaces can particularly act as ultraviolet nanolasers at room temperature. Furthermore, they reported the development of a novel microfluidic assisted nanowire integration (MANI) method for hierarchically assembling the building blocks of nanowires into desired functional systems and devices [66] (Figure 1).

##### Graphene Engineered Field-Effect Biosensors

Due to its special nanostructure, excellent optical and electrical properties, and good biocompatibility, graphene has rapidly become a hot material in biosensor research and has successfully detected a variety of small biological molecules, DNA, enzymes, proteins, and cells. Detecting DNA hybridization is critically dependent upon the scalable production of all-electronic DNA biosensors. The current developed fabrication approach enables the production of label-free DNA biosensors in a highly reproducible and scalable manner with >90% yield. These biosensors were based upon graphene field-effect transistors (GFETs) that had been functionalized using single-stranded probe DNA. GFET sensor Dirac point voltage varied in proportion to target DNA concentrations on these biosensors, which had a 1 fM limit of detection for 60-mer DNA oligonucleotides. It additionally confirmed that mismatch positioning altered the strength of DNA hybridization in their control studies. Overall, these highly sensitive DNA biosensors offer promise as an approach to rapidly and accurately detecting DNA hybridization and cost-effectively conducting sequencing. 

**Figure 2:**
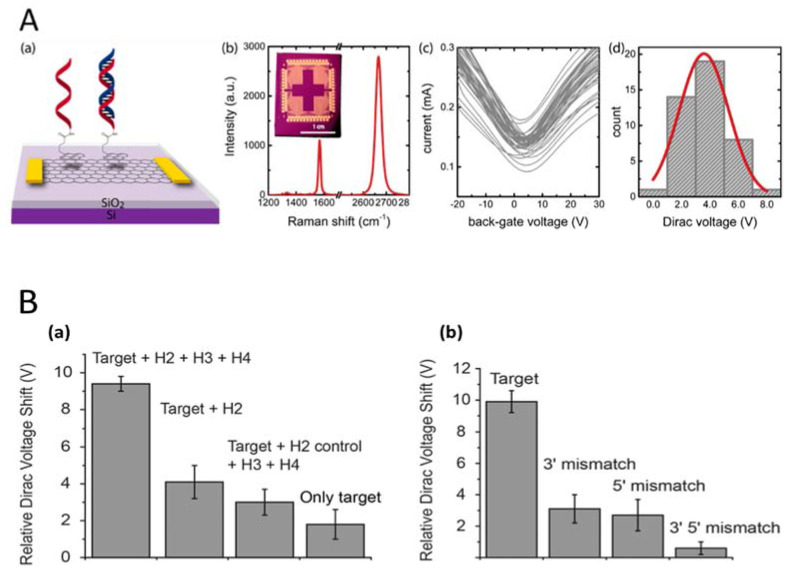
(**A**) (**a**) Schematic of DNA biosensor based upon a graphene field-effect transistor funct-ionalized with complementary probe DNA. (**b**) Raman spectrum of the channel region of a graphene field-effect transistor (GFET) after processing. Inset: Optical micrograph of an array of 52 GFETs. (**c**) I-Vg characteristics for an array of 52 GFET devices showing excellent reproducibility. (**d**) Histogram of the Dirac voltage extracted from the I-Vg characteristics of the panel (**b**) along with a Gaussian fit to the data (red curve) [70]. Reprinted with permission from ref. [67]. 2016 American Chemistry Society. (**B**) Biosensor response to positive and negative controls. (**a**) Relative Dirac voltage shifts for various positive control experiments based upon concentrations of 1 μm for the target DNA and the specified helper DNAs. (**b**) Relative Dirac voltage shifts for 10 nm target DNA and negative controls with base mismatches at the ends; detailed DNA sequences are listed in Table S1. Error bars are the standard deviation of the mean. Helper DNAs were 1 μm. Reprinted with permission from ref. [65]. 2018 American Chemistry Society.

Johnson’s group [65] reported a method to overcome the length-dependent property of the GFET DNA corrosivity sensor which depends on single-strand DNA testing. The test showed that compared with the traditional single-strand DNA testing, the clamp DNA testing has a higher specificity for incomplete DNA. A large area of graphene (10 cm × 15 cm) was incorporated into the copper foil through the low-pressure mixing smoke affidavit and transferred to the Si/SiO_2_ substrate through the recently manufactured Cr/Au anode. GFET DNA was functionalized by incubation with 1-pyrene butyric acid (base) in N, N-dimethylformamide (DMF). After functionalization, GFETs were incubated with a water sample array of aminated DNA fragments. Hairpin test DNA is metastable and can be opened explicitly by the target DNA, thus triggering the self-aggregation reaction. In the sodium citrate (SSC) hybrid vector, GFET biosensor clusters were mixed with known target DNA in the presence of H 2, H 3, and H 4 (all with a convergence of 1 μm), and I-Vg properties were estimated in the dry state. To explain the factors of self-polymerization amplification, a scientific model is established, which reflects the key biochemical reaction, which connects the target DNA oligomer to the start of an amplification reaction mediated by cycling H1 and H2. The model also anticipates that exploratory dose-response bending is an element of incubation time: at low targets (fm-run), considering that the target DNA reused will open additional H1 bands over time, thus increasing the number of H1 · H2 · H3 · H4 buildings, it is expected that Dirac voltage movement will develop if the test is conducted in a longer time (>1 h). The model is expected to be approved temporarily. Under the same experimental conditions, the incubation time was extended to 100 h instead of 1 h by three methods (100 pm, 100 fM, 100 am). The biosensor was immersed in 1 h and the polymerization degree was 100 PM. For the lower focal length (100 fM, 100 am), the reaction will gradually expand after a while. To verify the feasibility of identification methods based on target reuse and self-aggregation enhancement, GFET experiments were carried out on some positive controls [65,67] (Figure 2).

By contrast with the above work, the Long group developed a GFET biosensor that could be used to detect alanine aminotransferase (ALT) under a low operative voltage in a single reaction step. Chemical vapor deposition was used to fabricate this device using large-area thin graphene films. The resultant biosensors operated with a 0.1 V drain voltage and −0.1 to 0.3 V sweep gate voltage and exhibited significant pH sensitivity (23.12 mV/pH), a low hysteresis voltage (1.2 mV), and a small drift rate (4.74 mV/h). The authors were able to successfully detect ALT based upon the hybrid configuration of l-alanine and α-ketoglutarate immobilized on the graphene membrane, achieving good linearity (R^2^ = 0.99) in the 10–100 U/L ALT concentration range. Detection of cancer in its early stages is associated with better patient outcomes, and miRNAs represent excellent biomarkers that can be leveraged for early-stage cancer detection [68].

The Zhang group designed a gold nanoparticle (AuNP)-decorated GFET biosensor that was able to accurately, sensitively, and selectively detect specific miRNAs in a label-free manner. This biosensor was fabricated via drop-casting a reduced graphene oxide (R-GO) suspension onto a sensor surface, after which AuNPs were applied to this R-GO surface. Next, a peptide nucleic acid (PNA) probe was affixed to this AuNP surface, and PNA-miRNA hybridization was then used as a means of detecting miRNAs in sample solutions. This biosensor had a 10 fM limit of detection and was able to effectively differentiate between target miRNAs of interest, miRNAs bearing a single mismatched base, and non-complementary miRNAs. The authors were able to use this biosensor to detect miRNAs in serum samples while maintaining high sensitivity and selectivity, underscoring the diagnostic potential of these biosensors for the detection of early-stage cancer and other diseases associated with miRNA dysregulation [69].

##### MoS_2_ Nano-Interface

**Figure 3:**
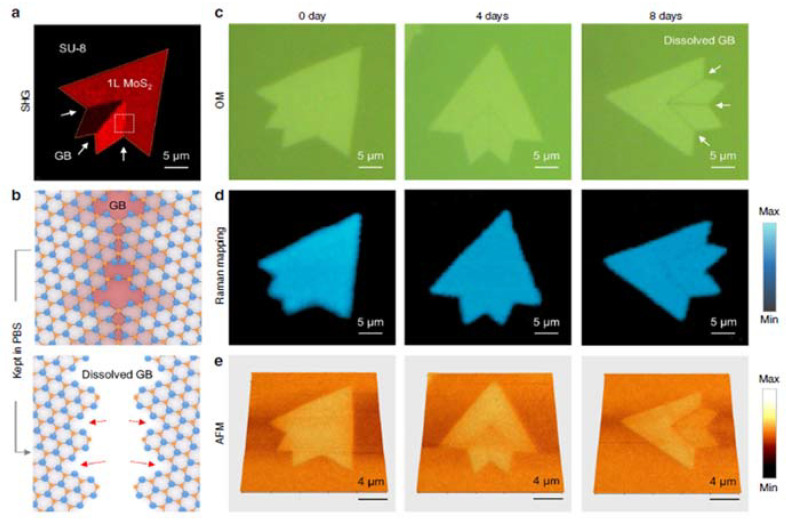
Morphological and structural evolution of isolated monolayer MoS_2_ crystals with different dissolution times in phosphate-buffered saline (PBS) solution. An image of an APCVD-grown polycrystalline monolayer MoS_2_ crystal on a SU-8/sapphire substrate, showing the distribution regions. (**b**) Schematic illustration of the MoS2 GB marked in the dotted box of (**a**) before and after dissolution in PBS solution. (**c**–**e**) OM (**c**), Raman A1g intensity mapping (**d**), and atomic force microscopy (AFM) (**e**) images of monolayer MoS_2_ crystals, immersed in a PBS solution (pH 7.4) at 75 °C with different dissolution times (0–8 days) [. Reprinted with permission from ref. [71]. 2019 Springer.

Typically, bulk 3D structures of FET biosensors presented the low sensitivity, which could be overcome by using 1D or 2D structures (nanotubes/nanowires), the latter face severe fabrication challenges, impairing their practical applications. As mentioned above, graphene is a promising 2D material for biosensors, currently, MoS_2_-based FET biosensors were reported the compatible sensitivity of that based on graphene. MoS_2_ is advantageous for biosensor device scaling without compromising its sensitivity. Additionally, MoS_2_ is highly flexible and transparent which offers opportunities in medical prostheses. Moreover, the theoretical analysis demonstrated that MoS_2_ was highly advantageous in biosensor device scaling without altering its sensitivity and capability for single molecular detection. The authors concluded that the high flexibility and transparent nature of MoS_2_ could be exploited for several novel opportunities in the field of diagnosis and medical prostheses. The results show that a combination of these unique characteristics makes MoS_2_ an extremely potential candidate for the fabrication of low-cost next-generation biosensors [71] (Figure 3).

**Figure 4:**
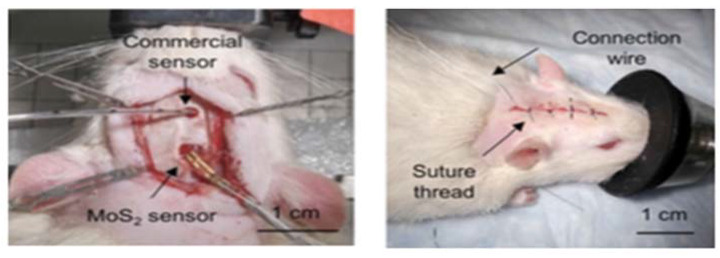
Optical images of a MoS_2_-based bioabsorbable sensor implanted in a rat together with a commercial one before and after suture. Reprinted with permission from ref. [71]. 2019 Springer.

To uncover the science and concoction energy of disintegration of monolayer MoS_2_ in a phosphate-buffered saline (PBS) arrangement, the Chen bunch originally explored secluded precious stones with few grain limits. The general end is that the disintegration of monolayer MoS_2_ precious stones in PBS happens as an imperfection actuated scratching process. In this work, the disintegration qualities and conduct of both confined precious stones and constant films of CVD-developed monolayer MoS_2_ in PBS arrangements at different temperatures and with various pH levels. Chen’s collective studied the cytotoxicity of MoS_2_ during the disintegration process, using the starting material of the continuous film, the separated chip, and the pre decomposition in a PBS arrangement, and then analyzed the long-range biocompatibility of MoS_2_ membrane in vitro. As well as the non-toxic nature of instantaneous MoS_2_ bioelectronics, the rationality of its long-term use in the human body is recommended. These findings of the biocompatibility and bioabsorbability of MoS_2_ make it applied in implantable and short biomedical sensors. Chen collected and described many MoS_2_-based bioabsorbable sensors, which are used to accurately estimate weight, temperature, strain, and motion. The sensors implanted in the intracranial space of the biological model (mouse) allow displaying the important utilization methods of observing patients in the rehabilitation period after a terrible mental injury. The results show that the results of continuous in vivo examination and traditional small tools of organic markers by fully bioabsorbable sensors (Figure 4).

The Banerjee group has demonstrated FET biosensors based on 2D molybdenum disulfide (MoS_2_), which provides high sensitivity and easy patternability. The MoS_2_-based pH sensor can achieve sensitivity as high as 713 for a pH change by 1 unit. Specific protein sensing is achieved with a sensitivity of 196, even at 100 femtomolar concentration [26]. According to the Banerjee group, FETs based on 3D structures are associated with lower sensitivity towards the analytes. Thus, alternative methods should be explored for addressing the issues of lower sensitivity associated with 3D structures. One-dimensional (1D) structures such as nanowires and nanotubes efficiently address the issue of lower sensitivity towards the target analytes; however, they face certain challenges during their fabrication which in turn limit their practical applications for biosensing. Owing to the 2D structure of the designed FET biosensor based on MoS_2_, it offered easy patternability and device fabrication and exhibited high sensitivity. The sensitivity of MoS_2_-based pH sensors was determined by performing the analysis in the range of pH 3–9 and the described pH sensor achieved elevated sensitivity of 713 with a change of pH by 1-unit in a wider pH range of 3–9. Furthermore, the fabricated sensor was also used for sensing specific proteins, resulting in its ultrasensitive sensing. At a low concentration of 100 femtomolar concentration, a sensitivity of 196 was achieved for the specific protein used as the analyte. The authors also compared the efficiency of their MoS_2_-based FET biosensor with graphene, a 2D material. They found that their MoS_2_-based FET biosensor exhibits almost 74-folds higher sensitivity as compared to graphene [26].

**Figure 5:**
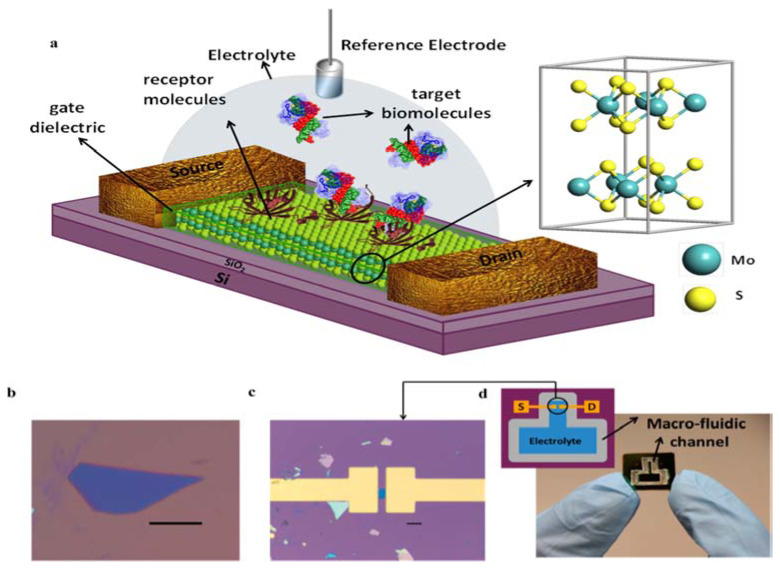
MoS_2_-based field-effect transistor (FET) biosensor device. (**a**) Schematic diagram of MoS_2_-based FET biosensor. For biosensing, the dielectric layer covering the MoS_2_ channel is functionalized with receptors for specifically capturing the target biomolecules. The charged biomolecules after being captured induce a gating effect, modulating the device current. An electrolyte gate in the form of an Ag/AgCl reference electrode is used for applying the bias to the electrolyte. The source and drain contacts are also covered with a dielectric layer to protect them from the electrolyte (not shown in this figure). (**b**) Optical image of a MoS_2_ flake on 270 nm SiO_2_ grown on degenerately doped Si substrate. Scale bar, 10 μm. (**c**) Optical image of the MoS_2_ FET biosensor device showing the extended electrodes made of Ti/Au. Scale bar, 10 μm. (**d**) Image and schematic diagram (inset figure) of the chip with the biosensor device and microfluidic channel for containing the electrolyte. Inlet and outlet pipe for transferring the fluid and the reference electrode are not shown in the figure. Reprinted with permission from ref. [26]. 2014 American Chemistry Society.

To advance this technique, the Yu group [72] reported label-free highly sensitive FET biosensors based on 2D MoS_2_ avoiding additional absorption layers. Fibroblast growth factor 21 (FGF21) is considered a vital biomarker and has been reported to be used in the early detection of non-alcoholic fatty acid liver disease (NAFLD). Currently, this growth factor is getting wider scientific attention and efforts have been made to develop the most effective method to detect its extremely low-level in the biological system. They investigated the biosensor for the detection of FGF21 present in a non-aqueous environment. Through immobilization on the surface of MoS_2_, the designed biosensor was capable of detecting as low as 10 fg mL^−1^ of the target FGF21. Multiple sets of parallel experiments were conducted to demonstrate the consistency and reproducibility of MoS2 FETs. Moreover, the biosensor was also highly sensitive to detect FGF21 in complex samples (i.e., serum sample), authenticating its potential to be used in NAFLD diagnosis. Generally, this study demonstrated the alternative platform based on thin-layered transition-metal dichalcogenides (TMDs) for highly selective and sensitive novel biosensors development [26] (Figure 5).

#### 2.1.2. Membraneless Field-Effect Transistor (FET) Biosensors

**Figure 6:**
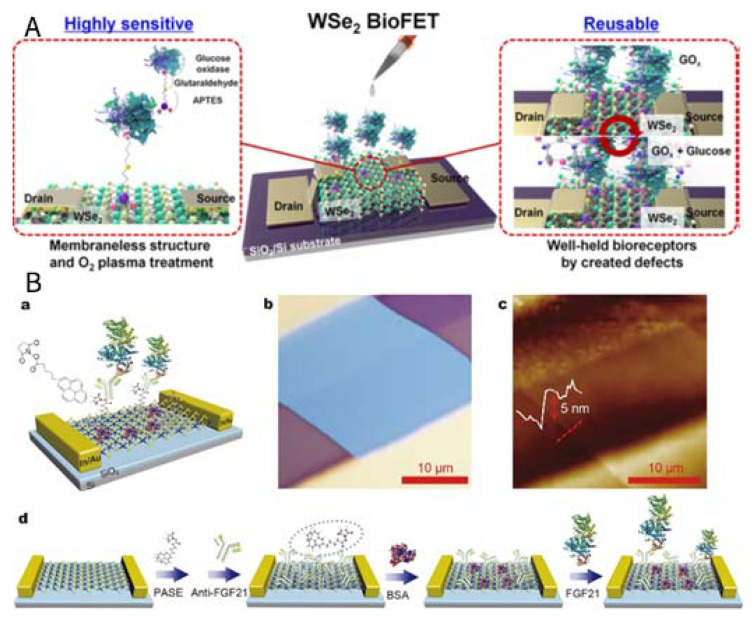
(**A**) Membraneless FET-type tungsten diselenide (WSe_2_) biosensors Reprinted with permission from ref. [19]. 2018 American Chemistry Society (**B**) (**a**) Schematic representation of the label-free MoS_2_ FET as a biosensor to electrically detect FGF21. (**b**) Optical and (**c**) AFM images of the MoS_2_ FET transistor. Inset in (**c**) shows the MoS_2_ thickness. (**d**) Experimental process of surface chemistry to prepare anti-FGF21 on MoS_2_ surface and its reaction with FGF21 Reprinted with permission from ref. [71]. 2019 Springer.

The membraneless FET can be used as an alternative platform for developing electrical biosensors with high sensitivity. They have received considerable attention in their efforts to develop an effective method that detects low levels of the biomarker. FET-type biosensors (BioFETs) have been a topic of increasing interest in recent years owing to their ability to detect analytes of interest in a selective, sensitive, rapid, and label-free manner, and owing to the fact that these biosensors are amenable to miniaturization. To date, however, the sensitivity of BioFETs has been limited by their need for an insulating membrane that prevents nonspecific binding from occurring as a consequence of dangling surface bonds associated with typical semiconductors. The Park group [19] developed a reusable, sensitive, and membrane-free BioFET by leveraging tungsten diselenide (WSe_2_) as a defect-free van der Waals material. By using oxygen plasma treatment to generate a limited number of surface defects, the authors were additionally able to incorporate additional bioreceptor immobilization binding sites on the surface of these BioFETs, thus increasing their sensitivity to 2.87 × 10^5^ A/A for 10 mM glucose. Even after several rounds of glucose application and subsequent washing, these WSe_2_-based BioFETS maintained their excellent sensitivity (Figure 6).

Parker’s gathering showcases an extremely refined, reusable, film-free biofilm of tungsten disselenium (WSe_2_), one of the channeled materials, WSe_2_, which has a layered structure of progressive metal dihalides dependent on van der Waals (VDW) interactions. There is no catenary bond deformation on the surface of WSe_2_, considering the deep touching activity of the membraneless WSe_2_ biofilm. It is not subject to fuzzy authority. They extended the falsifiability of Wse_2_ biofilms by generating a small number of defects on the non-defective surface as a restrictive destination for the biological receiver. They treated WSe_2_ slices of silicon dioxide/silicon substrate with oxygen plasma for 15 and 30 s. As indicated by XPS results, aggregation speculates that O_2_ plasma treatment destroys the security of w-Se, thus generating Se opportunities and w-o bonds on the surface of WSe_2_. The biological receptor (glucose oxidase: GO) was fixed on the surface after WSe_2_, O_2_ plasma treatment. We arranged the control, 15 and 30 s plasma treatment WSe_2_ test and incubated the example in the APTES (3-aminopropyl triethoxysilane) response for 30 min. Fill in the form of a synthetic linker to anchor the gox biosector atoms to the WSe_2_ biosensor. The membrane-free WSe_2_ biofilm was tested and rinsed with glucose more than once to analyze its reusability [19] (Figure 6).

(3) $$Glucose\;\left(C_{6}H_{12}O_{6}\right)+Glucose\;oxidase_{\left(ox\right)}\to gluconolactone\left(C_{6}H_{12}O_{6}\right)+glucose\;oxidase_{\left(red\right)}$$

(4) $$Glucose\;oxidase_{\left(red\right)}+O_{2}\to glucose\;oxidase_{\left(ox\right)}+H_{2}O_{2}$$

The group of Yu group reported label-free highly sensitive FET biosensors based on 2D molybdenum disulfide (MoS_2_) avoiding additional absorption layers. They investigated the biosensor for the detection of FGF21 present in a non-aqueous environment. Through immobilization on the surface of MoS_2_, the designed biosensor was capable of detecting as low as 10 fg mL^−1^ of the target FGF21. Multiple sets of parallel experiments were conducted to demonstrate the consistency and reproducibility of MoS_2_ FETs. Moreover, the biosensor was also highly sensitive to detect FGF21 in complex samples (i.e., serum sample), authenticating its potential to be used in NAFLD diagnosis. Generally, this study demonstrated the alternative platform based on thin-layered transition-metal dichalcogenides (TMDs) for highly selective and sensitive novel biosensors development [72].

### 2.2. Photoelectrochemical Biosensors

In photoelectrochemistry, light is utilized to produce electron/opening sets in a photoactive material, and these electron/gap sets, when isolated, are utilized to drive redox responses. Contingent upon the responses happening in the photoelectric compound (PEC) cell, light is then changed over to electrical or synthetic energy. Recently, PEC signal transduction has been exhibited for organic detecting. In PEC biosensors, light is utilized to create charge transporters in photoactive materials, and the transduced electrochemical current is estimated for examining naturally significant targets. Since signal readout is electrochemical, this technique acquires the advantages of electrochemical biosensing:

1. The sign is perused utilizing economical and simple to-utilize instrumentation.
2. Multiplexed location is accomplished utilizing multielectrode microchips.
3. Due to optical excitation, PEC estimations are performed at lower inclination possibilities contrasted with their electrochemical partners.
4. This brings down the deliberate electrochemical foundation flows and builds the sign-to-foundation proportion. PEC readout has been utilized to identify biomolecules, for example, DNA, RNA, and proteins. In any case, when utilizing these creation techniques, the compromise must be made between the level of auxiliary tunability, throughput, and cost.

**Figure 7:**
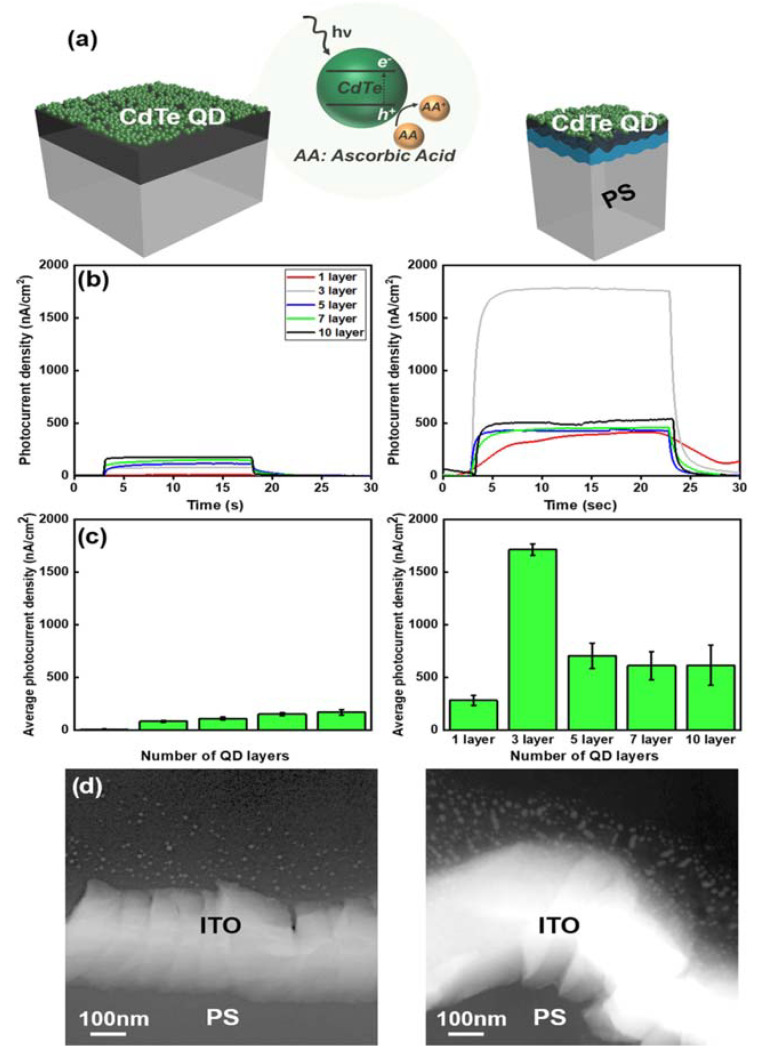
Photoelectric compound (PEC) measurements at planar and scaffolded-wrinkled photoelectrodes. (**a**) Visible light (470 nm) induces electron/hole pairs in CdTe quantum dots (QDs). Holes oxidize ascorbic acid, generating an anodic redox current. (**b**) PEC current densities measured on planar (**left**) and scaffolded-wrinkled (**right**) devices using 100 mM ascorbic acid at 0 V with respect to Ag/AgCl. The QD layers are increased by depositing alternate layers of QDs and poly (diallyl dimethylammonium chloride) (PDDA). The 470 nm light-emitting diode is turned on at 3 s and turned off at 23 s. (**c**) The average PEC current densities measured on planar (**left**) and scaffolded-wrinkled (**right**) devices for different numbers of QD layers. (**d**) Scanning transmission electron micrographs of cross-sections of planar (**left**) and scaffolded-wrinkled (**right**) devices. The imaged devices contain three QD layers Reprinted with permission from ref. [20]. 2018 American Chemistry Society.

The gathering utilized surface wrinkling to upgrade the proficiency of photocurrent age in detecting PEC cells. Surface wrinkling is a simple and economical technique for bringing tunable smaller scale and nanostructuring into dainty films, permeable systems, and get together of nanoparticles. In this work, The Soleymani group devised a strategy for stacking photoactive quantum dots (QDs) into a wrinkled platform of a straightforward conductive oxide to improve the produced photocurrent (Figure 7). By functionalizing the photoactive QDs implanted in the wrinkled film, we built up a sensor for distinguishing DNA targets. The wrinkles were made legitimately on polystyrene by shaping a hardened oxidized surface layer and contracting the substrate. The indium tin oxide (ITO) and QDs were then sputtered and stacked into the wrinkled polystyrene framework, separately. The distinctions in surface geology and progression between the three classes of photoelectrodes. The standard photoelectrodes are planar, and their ITO layer was free of breaks. These components, which the article talked about, additionally empowered the oxidized layer to adjust instead of break in light of the applied strain, making a continuous wrinkled layer, and afterward, PEC current for the scaffolded-wrinkled surface (at 3 layers) was around multiple times bigger than the current accomplished in the planar surface. The stem was utilized to think about the PEC current pattern and as the charges travel through the system of QDs and the PDDA (poly (diallyl dimethylammonium chloride)) spacer, resistive misfortunes, dispersion misfortunes, and recombination decline their gathering rate by the ITO anode, further lessening the photograph current. The gathering explored the progressions in PEC current when the scaffolded-wrinkled photoelectrodes were interfaced with test and target DNA strands. The upgraded PEC current acquired utilizing the wrinkled materials design empowered us to build up a delicate and mark-free DNA biosensor with a picomolar limit-of-location [20].

**Figure 8:**
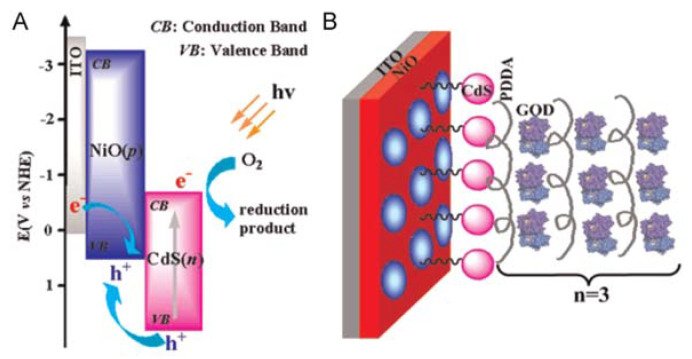
(**A**) Schematic diagrams for energy bands of n-CdS and p-NiO and photocurrent generation mechanism of NiO/CdS in the presence of oxygen. (**B**) The assembly of glucose oxidase (GOD) on indium tin oxide (ITO)/NiO/Cd Selectrode. Reprinted with permission from ref. [69]. 2014 ScienceDirect.

Zhang et al. [69], reported the design and development of a self-operating photocathode which was based on the CdS quantum dots sensitized with 3D nanoporous-NiO (Figure 8). The photocathode reversibly exhibited high selectivity towards dissolved oxygen (working as electron donor) in the electrolyte solution. Employing the photocathode, a novel photoelectrochemical sensor was developed for the detection of glucose while using glucose oxidase (GOD) as a biocatalyst. The use of various competing substances such as dopamine (DA), cysteine (Cys), ascorbic acid (AA), and H_2_O_2_ revealed that they did not affect the cathodic photocurrent of the 3D NiO/CdS electrode. However, they significantly influenced the photocurrent of the photocathode, thus authenticating the selectivity of the described method.

The applicability of the method was investigated for the detection of glucose present in real samples including glucose injection and blood serum. The obtained results showed they were highly satisfactory in terms of accuracy. The authors of the study concluded that these findings could open new avenues for designing and developing photochemical biosensors based on self-operating photocathode, and this would, in turn, promote the applications of semiconductor nanomaterials in photoelectrochemistry. The further report showed the design and development of a self-operating photocathode which was based on the CdS quantum dots sensitized with 3D nanoporous-NiO. The photocathode reversibly exhibited high selectivity towards dissolved oxygen (working as electron donor) in the electrolyte solution. Employing the photocathode, a novel photoelectrochemical sensor was developed for the detection of glucose while using glucose oxidase (GOD) as a biocatalyst. The use of various competing substances such as dopamine (DA), cysteine (Cys), ascorbic acid (AA), and H_2_O_2_ revealed that they did not affect the cathodic photocurrent of the 3D NiO/CdS electrode. However, they significantly influenced the photocurrent of the photocathode, thus authenticating the selectivity of the described method [69].

**Figure 9:**
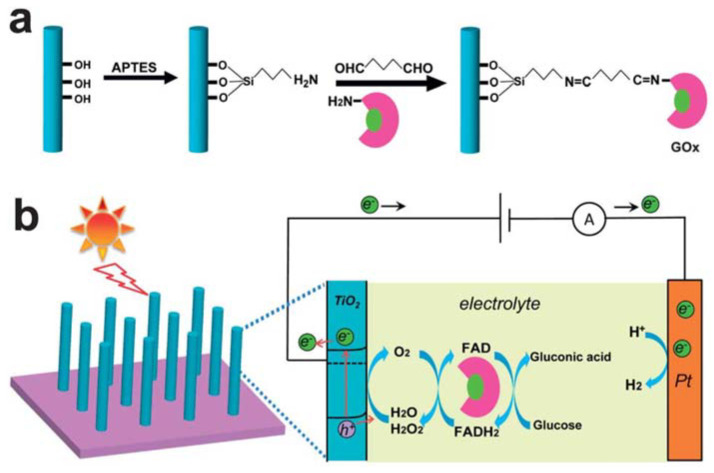
(**a**) Schematic of glucose oxidase (GOx) functionalization on the TiO_2_ nanowire surface via silane/glutaraldehyde linkage chemistry. APTES: 3-aminopropyl triethoxysilane. The green and pink colors of GOx indicate the flavin group (FAD) redox center and the external insulating protein shell, respectively. (**b**) Schematic of the TiO_2_–GOx NW-based PEC sensor for glucose detection. Under sunlight illumination, the photogenerated holes oxidize H_2_O or H_2_O_2_ over the TiO_2_NW anode to form O_2_, which serves as an efficient electron acceptor of FAD/FADH2. Glucose is oxidized to gluconic acid during this cycle. On the cathode (Pt), the photogenerated electrons reduce water to form H_2_. The current flowing through the circuit is recorded as the sensing signal. Reprinted with permission from ref. [73]. 2013 The Royal Society of Chemistry.

Zheng et al. [73] reported the synthesis of a novel sensor for the detection of glucose (Figure 9). The sensor was based on single-crystalline TiO_2_ nanowires that were synthesized through a hydrothermal synthetic route. The nanowires were further surface-functionalized with glucose oxidase, and enzymes responsible for the oxidation of glucose to gluconic acid. The development of the sensor was based on the photogenerated holes on the TiO_2_ anode surface that could generate O_2_. Thus, the O_2_ was considered responsible for the efficient shuttling mediator of electron between the surface of the sensor and the redox center of glucose oxidase. This in turn resulted in significant photocurrent enhancement. The surface-functionalized nanowire-based sensor was found to be highly sensitive towards glucose in buffer with an as low as ~0.9 nM lower limit of detection. The sensor was further investigated for its glucose detection capability in complex system mouse serum. The authors concluded the novel nanowire-based photoelectrochemical sensor as an efficient, convenient, and cost-effective diagnostic for the detection of disease biomarkers [73]. The Tang report that PEC biosensors have been used for mycotoxin detection due to their properties. Photoactive materials like this have important roles in converting chemical information into detectable PEC signal. Specific recognition elements also affect the analytical performance of PEC biosensors [74].

Photoelectrochemical detection of analytes is greatly preferred as it presents an interesting signal transduction modality. Modulation of the charge carriers in such sensing systems occurs as a result of the redox reactions of molecular targets that take place on the surface of the electrode. Owing to the superior properties of PEC biosensors, they are considered to be used as a promising tool for the detection of mycotoxin. When employed in the fabrication of biosensing systems, photoactive materials work as transducers, thus they convert the chemical information into detectable signals. The analytical performance of biosensors based on PEC materials is greatly influenced by the signal strategies of the specific recognition element. A review by Tang et al. extensively reviewed the photoactive materials and their signal strategies employed for the fabrication of PEC biosensors used for the detection of mycotoxin. They also discussed the future aspects of these biosensors in detail.

### 2.3. Nano Optical Biosensor

Nanoscale optical biosensors are currently attracting greater scientific interest due to their easy fabrication, characterization, and cost-effectiveness. The Duyne group [75] reported the usage of nano-sphere lithography (NSL) for the fabrication of gold and silver nano-triangles and the resulting localized surface plasmon resonance (LSPR) spectra were recorded by using ultraviolet (UV)–Visible extinction spectroscopy. They found that the short-range distance dependence (almost 0–2 nm) of electromagnetic fields surrounding the resonantly excited nanoparticles can be modulated through varying their structure, composition, and size. The measurement was based on shifting of LSPR spectra wavelengths (*ì*max) that appeared due to the absorption of hexadecane-thiol as a function of nanoparticle shape (truncated tetrahedron versus hemisphere), size (in-plane width, out-of-plane height, and aspect ratio) and composition (silver versus gold). It was observed that hexadecane-thiol-induced LSPR shift for silver triangles decreased with an increase in width (in-plane) at a fixed height (out-of-plane) or vice versa. The described trends were in contrast to the findings of the previously published report which studied long-range distance dependence examining layers with 30 nm thickness [75]. However, the confirmation of these results for both short and long-range analysis was based on theoretically using finite element electrodynamics. Findings of the study revealed that short-range distance-dependence results were found sensitive to hot spots (these are the regions having highly induced electric fields) located in peripheries of the triangles, and hence they suggested the involvement of enhanced local fields for the generation of extinction spectra. The results also exhibited the appearance of larger hexadecane thiol-induced LSPR peak shifts in the case of nano triangles as compared to hemispheres of the same volume. Similarly, a larger peak shift was observed for the silver nano-triangles as compared to gold nano-triangles having the same out-of-plane heights and in-plane widths. It was also found that alkane-thiol-induced LSPR peak shift of chain-length present in silver nano triangles was independent of the size. A better understanding of the reported short-range dependence of the adsorbate-induced LSPR peak shift associated with nanoparticle composition as well as structure will provide knowledge to improve the sensitivity of nanosensors based on refractive-index [76].

**Figure 10:**
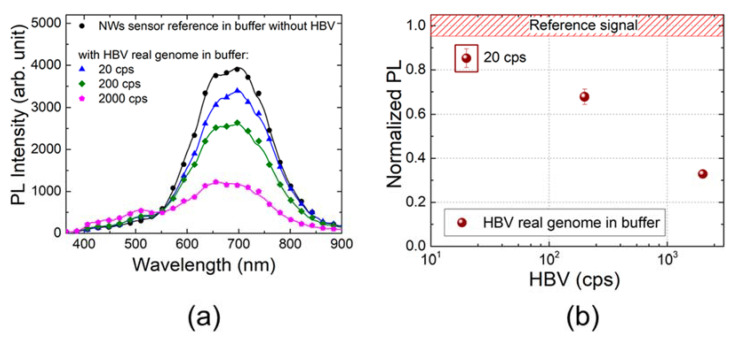
(**a**) Photoluminescence (PL) spectra of the NWs sensor tested in hepatitis b virus (HBV) real genome extracted from infected human blood and spiked in buffer reported for different concentrations ranging from 20 cps up to 2000 cps. The PL reference of the sensor without any copies of HBV is shown in black. (**b**) The trend of the PL integrated peak of the deconvolved NWs PL emission as a function of real HBV genome concentration normalized to its reference signal (red bar) obtained by the buffer solution without any real HBV copy. Reprinted with permission from ref. [77]. 2018 American Chemistry Society.

Nano-silicon photonics is an ideal stage to realize high orthodontic and selective recognition of organic atoms under complex fluid conditions. In this case, luminescent silicon-based nanostructures are very encouraging materials because their large uncoated surfaces and their optical properties are due to the most innovative conduction techniques. In the aspect of DNA recognition, the basic detection of Si-nws was carried out by using the electric conduction strategy based on DNA hybridization conductance variation and the explicit detection method fixed on the surface of nanowires (NWs), and 220 atoms (about 6600 DNA target duplicates per 50 mL) that were low fracture points were found. SSDNA, by using a variety of crystal silicon nano-FETs with measurements of less than 20 nm, another interesting approach relies on fluorescent labeling of DNA. In particular, his collection demonstrates the high performance of Si NWs biochemical sensors to reduce the identification of various named genome sequences to as low quality as possible. Sabrinas’ [77] collections reported major cases of direct genomic identification in Si-NWs optical biosensors without enhancement steps (sans-pcr) and markers (unlabeled) (Figure 10). The proposed approach utilizes a hybrid approach that combines Si NWS with satisfactory in situ hybridization from two explicit experiments and synthesizes it on the surface with a genomic double strand. We tried to demonstrate sensors that take advantage of the hepatitis b virus (HBV) genome. Figure 10a the concentrations range from 20 cps to 2000 cps for the Photoluminescence (PL) spectra of the NWs sensor that was tested in HBV genome extraction from infected human blood. The PL reference of the sensor without any copies of HBV is depicted in black. Figure 10b shows that the PL integrated peak of the NWs PL emission is obtained by the buffer solution without any real HBV copy. Lastly, the PL signal is used as a detection mechanism.

The impact of a natural network and its potential impedances can be evaluated by testing the Si NWs sensor with HBV clone genome broke up in human serum rather than the support arrangement. To affirm this theory, a Si wafer (without NWs) was tried with an answer made by human serum without HBV, and a similar wide multipeaked band 400–600 nm was watched and verified that the PL signal variety of the NWs sensor as a component of HBV focus is the equivalent in the two grids. The presentation evaluation of the Si NWs sensor with genuine examples is a significant point to be tended to. To further explore this point, we tried the gadget by utilizing a genuine HBV genome removed from a blood test. It was affirmed that this Si NWs sensor can distinguish the genuine HBV genome separated from human blood with an effectiveness similar to the constant PCR (20 cps/response), regardless of whether its length is about the portion of the analytical sample [78].

The Duyne group reported the usage of NSL for the fabrication of gold and silver nano-triangles and the resulting LSPR spectra were recorded by using UV-Visible extinction spectroscopy. They found that the short-range distance dependence (almost 0–2 nm) of electromagnetic fields surrounding the resonantly excited nanoparticles can be modulated through varying their structure, composition, and size. The measurement was based on shifting of LSPR spectra wavelength (*ì*max) appeared due to the absorption of hexadecane-thiol as a function of nanoparticle shape (truncated tetrahedron versus hemisphere), size (in-plane width, out-of-plane height, and aspect ratio) and composition (silver versus gold). It was observed that hexadecane-thiol-induced LSPR shift for silver triangles decreased with an increase in width (in-plane) at a fixed height (out-of-plane) or vice versa. The described trends were in contrast to the findings of the previously published report which studied long-range distance dependence examining layers with 30 nm thickness [75]. However, the confirmation of these results for both short and long-range analysis was based on theoretically using finite element electrodynamics. Findings of the study revealed that short-range distance-dependence results were found to be sensitive to hot spots (these are the regions having highly induced electric fields) located in peripheries of the triangles, hence suggested the involvement of enhanced local fields for the generation of extinction spectra. The results also exhibited the appearance of larger hexadecane thiol-induced LSPR peak shifts in the case of nano triangles as compared to hemispheres of the same volume. Similarly, a larger peak shift was observed for the silver nano-triangles as compared to gold nano-triangles having the same out-of-plane heights and in-plane widths. It was also found that the alkane-thiol-induced LSPR peak shift of chain-length present in silver nano triangles was independent of the size. A better understanding of the reported short-range dependence of the adsorbate-induced LSPR peak shift associated with nanoparticle composition as well as structure will provide knowledge to improve the sensitivity of nanosensors based on refractive-index [76].

### 2.4. Metal-Assisted Interface

**Figure 11:**
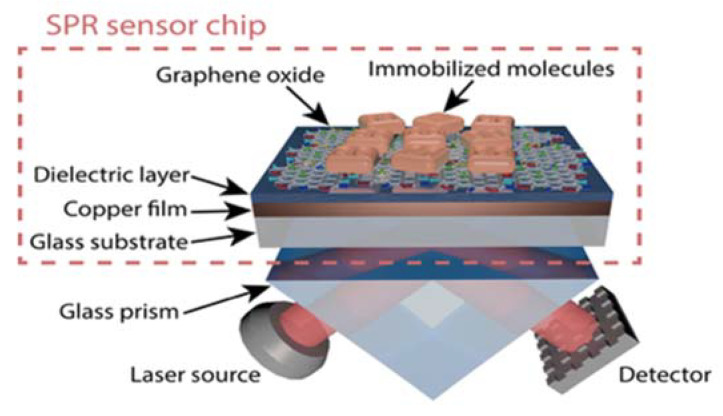
Schematic representation of the surface plasmon resonance (SPR) biosensor comprising the SPR sensor chip based on plasmonic copper films coated with a dielectric layer to protect against oxidation. The prism and sensor chip substrates are made of the same type of glass, which allows for an efficient optical connection. The immobilization of biomolecules on the biosensor surface can be achieved using a graphene oxide linking layer deposited atop the dielectric layer. Reprinted with permission from ref. [79]. 2018 American Chemistry Society.

**Figure 12:**
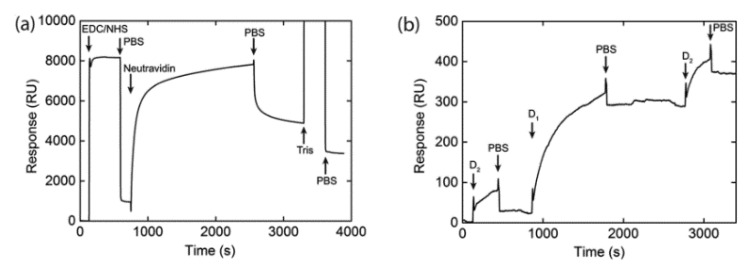
(**a**) Covalent immobilization of neutravidin on the surface of the graphene oxide (GO) linking layer deposited on the copper surface plasmon resonance (SPR) sensor chip protected by 15 nm thick Al_2_O_3_ film. The immobilization procedure includes the activation of carboxyl groups of GO by the mixture of 0.4 M 1-ethyl-3-(3-(dimethylamino) propyl) carbodiimide hydrochloride (EDC) and 0.1 M N-hydroxysuccinimide (NHS) solutions and deactivation of carboxyl groups after neutravidin adsorption by 1 M Tris solution. (**b**) Adsorption of oligonucleotides D1 and D2 on the surface of neutravidin–GO copper SPR chip. D1 is biotinylated and complementary to non-biotinylated D2. PBS: phosphate-buffered saline Reprinted with permission from ref. [79]. 2018 American Chemistry Society.

Plasma biosensors are widely used in logic and medicine research, medical diagnosis, veterinary practice, nutrition, and health control. Stebunovs et al. [79] collects copper as a plasma material for constructing biosensor interfaces.(Figure 11) One potential way to overcome this problem is to protect the hidden metal surface with a graphene-covered barrier while having a negligible effect on the optical properties of the interface between the sensor surface and the substance to be tested or the organic frame. Stebunovs’ team proposed the SPR sensor chip, which relies on plasma-copper membranes fixed with different dielectric layers (Figure 12). The disappearance of the electron column is an essential part of the standard process, which stores thin copper film on the surface of the glass substrate. The optical properties of the metal film determine the probability of SPR excitation in a specific structure, the reverberation quality, and the feasibility of SPR biosensors. The ellipsoidal model shows that the dielectric constants of copper and gold films are directly determined by the ellipsoidal information. The variation of copper SPR sensor chips with different protective layers is hypothesized and preliminarily tested. Improvement with the ligand-specific interface in SPR biosensing applications analysis is important because 2D material has large surface area and different material properties, and the interface can be applied to a wide range of biological chemical association study, no matter under what circumstances, compared with sulfur connection layer, increasing the limit immobilized. This recent report showed the progress of the go connection layer covering the dielectric layer outside the SPR biosensor chip [78]. The Chang group proposed and validated a highly sensitive metal layer-assisted guide mode resonance (MaGMR) device for use in bioanalytical contexts. These researchers found that reflection spectra associated with this approach presented a unique inverse response, and were able to explore the underlying mechanistic basis for observed resonance. The high sensitivity of their MaGMR device was found to be attributable to its asymmetric resonance model profile and the low propagation angle inside the waveguide. Relative to typical GMR, these researchers observed a 1-fold enhancement of the evanescent waves within the analyte region. In experimental analyses, they found that the MaGMR was able to achieve a bulk sensitivity of 376.78 nm in fundamental TM mode while resonating at 0.809μm with the first diffraction angle. They observed a 264.78% enhancement in the sensitivity relative to typical GMR sensors under identical TM mode resonance conditions [80].

## 3. Perspectives and Future Direction

There have been key developments in the rapidly evolving field of organic electronics over the past two decades as these materials have increasingly been explored as an alternative to inorganic semiconductors. Owing to their ability to detect nanoscale materials, organic distributed feedback lasers represent a potentially ideal tool for sensing applications in biomedical and biological contexts. We note the current developmental and commercialization status of organic light-emitting diodes, photovoltaics, and thin-film transistors. Here we focus on a discussion of organic transistors and their recent application in the field of biosensor development. By presenting numerous examples and relevant citations, this will provide a comprehensive overview of the properties and principles of these biosensors.

### 3.1. Organic Semiconductor Interface for Biosensor

According to the specific material framework, various strategies are used to create extremely light crystalline layers, including various stripping methods and material manufacturing methods [81,82,83,84]. These programs play a very good role in most van der Waals layered materials, because the absence of a covalent bond between adjacent layers improves the strength of the mixture, and eliminates the problems caused by the hanging bond on the surface of the material so that the thickness of the “defect-free” gem is reduced to the core thickness. It is reported that the electronic properties of the materials can be quantitatively measured by the ion fluid gate field-effect transistor (FET) which is prepared on the large drop point gem.

**Figure 13:**
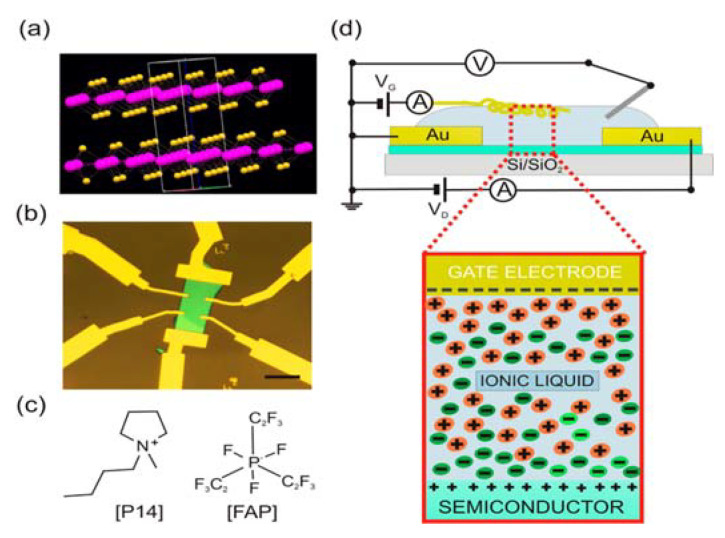
(**a**) Crystal structure of layered WS_2_; the purple balls correspond to W atoms and the yellow balls to S atoms. (**b**) Optical microscope image of a six-terminal Hall bar fabricated on a crystalline WS_2_ thin flake (the scale bar is 10 μm long). (**c**) Molecular structure of the ionic liquid employed as an electrolyte–gate dielectric. Both cation [P14]^+^ and anion [FAP]^−^ are shown. (**d**) Cross-section of a WS_2_ ionic liquid-gated FET. A large area of gold mesh and an oxidized Ag/AgO wire are used as a gate electrode and quasi-reference electrode, respectively. When the gate electrode is biased, two electrical double layers are formed at the gate/electrolyte and semiconductor/electrolyte interfaces, enabling charge accumulation at the semiconductor surface. Reprinted with permission from ref. [85]. 2012 American Chemistry Society.

Morpurgo’s group presented WS_2_ type sensor with ionic liquid as electrolyte. [85] (Figure 13) WS_2_ is a kind of ring gap semiconductor (ΔWS_2_ 1.35 eV), which consists of two-dimensional (2D) covalently enhanced s-w-slayer and van der Waals gap isolation (Figure 13). It is found that by coupling WS_2_ microchip with ionic liquid medium, stable, non-sluggish, electronic, and gap conductance adjustable bipolar transistors can be obtained. It can be imagined that the bandgap of WS_2_ can be reasonably determined with high accuracy (10%) by the dependence of the currently active channel’s input voltage, and the careful use of the ion fluid gate transistor provides a great asset for the quantitative study of the electronic characteristics of fragile gemstones. Slim crystalline pieces of WS_2_ were acquired by mechanical peeling of a mass single precious stone developed by synthetic fume transport and afterward moved on an exceptionally doped Si/SiO_2_ substrate. Chips appropriate for electrical portrayal were distinguished under an optical magnifying lens and their thickness was estimated by atomic force microscopy (AFM). Electrical contacts were manufactured by electron-bar lithography, trailed by metal (Ti/Au) dissipation and lift-off. The gadget yield attributes, estimated for the two polarities of VG and VD, affirm the high-caliber of the ambipolar transistor activity. The gathering can absolutely decide the bandgap of WS_2_ from basic vehicle estimations on a nanofabricated FET and ionic fluid gated FETs made on WS_2_ slim drops are demonstrated to be perfect ambipolar gadgets on account of an essentially ideal electrostatic coupling between the ionic fluid door and the transistor channel and to the high caliber of the utilized semiconductor. 

**Figure 14:**
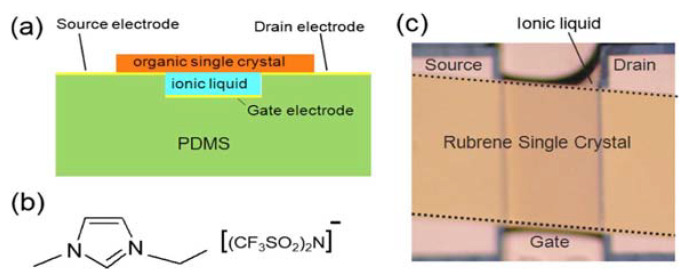
Color online (**a**) Structure of organic crystal/ionic liquid transistors. (**b**) [emim][TFSI]. (**c**) Optical view of a rubrene crystal/emimTFSI transistor [76]. Reprinted with permission from ref. [78]. 2012 American Institute of Physics.

Takeya’s work revealed another class of natural semiconductor gadgets functionalized by the quick ionic movement in the room temperature liquid salts [78]. In this work, strong to-fluid interfaces are framed between natural semi-conductor single gems and ionic fluids so the structures fill in as quick exchanging natural field-impact transistors, OFETs, with the most elevated transconductance gm = ID/VG per square channel sheet, i.e., the most proficient reaction of the yield channel current ID to the information door voltage VG among the OFETs so far constructed. OFETs offer the possibility for cutting -edge minimal effort de-indecencies that can be delivered by straightforward creation forms. There have been an account of the utilization of such polymer electrolytes as LiClO_4_ disintegrated in polyethylene oxide or ionic fluid continued in a polymer gel. However, these gadgets experience the ill effects of either more unfortunate versatility or moderate reaction to VG as a result of generally moderate ionic dispersion in the polymer grid. The OFETs most regularly comprise natural polycrystalline structure and dielectric protectors, for example, silicon dioxide. With common thicknesses of the door dielectric protectors of two or three hundred nanometers, many volts are expected to apply adequate EG for enough Q so down to earth flow intensification is accomplished. Takeya’s gathering use room temperature ionic liquids (RTILs) for the door protecting layer and natural single precious stones for the semiconductor layer (Figure 14). The electrical double layer (EDL) capacitance of 1-ethyl-3-methylimidazolium bis(trifluoromethylsulfonyl)imide [emim][TFSI] is estimated utilizing a test gadget comprising of the equivalent PDMS structure, where the top terminal is made out of gold slim films. The EDL capacitance of the RTILs stays huge even at 1 MHz, showing the quick ionic dispersion because of the voltage application. Thus, the EDL OFETs fusing the RTILs permit exchanging activity at such a high frequency. After presenting legitimate measures of RTIL, the fluid-filled in the gap is continued steadily by the capillary force. Owing to the high charge carrier mobility in rubrene single crystals and the high capacitance of EDLs in the electrolyte, the device realizes the highest transconductance ever achieved for organic transistors.

**Figure 15:**
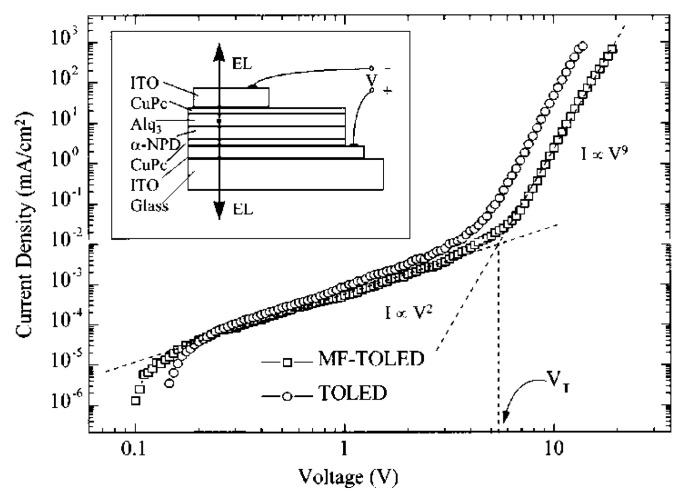
Current-voltage characteristics of a 0.4 mm diameter metal-free transparent organic light-emitting device (MF-TOLED) and TOLED has grown in the same vacuum cycle. Inset: schematic illustration of the MF-TOLED structure. Reprinted with permission from ref. [86]. 1998 American Institute of Physics.

The Forrest’s group [86] introduced a class of non-metallic cathodes with unique properties of high-transparency and low-reflectivity having useful applications in a variety of organic devices (Figure 15). The metal-free cathode was fabricated via employing thin copper phthalocyanine (CuPc) which was further surface coated with radio-frequency and low-power sputtered ITO film. The purpose of (CuPc) film was to protect the organic layers from possible damage during the process of ITO sputtering. A model was established which proposes that the damage-induced states at the interface of non-metallic cathode/organic film result in efficient electron injection properties of the cathode. Because of the low reflectivity of the metal-free cathode, a non-antireflection-coated, metal-free transparent organic light-emitting device (MF-TOLED) was developed having almost 85% visible transmission. Results showed that almost the same amount of light was emitted in both forward and backscattered direction. It was observed that the performance of MF-TOLED was almost identical to the performance of conventionally used TOLEDs which employ a cathode having significant reflective and absorptive characteristics based on the semitransparent thin film of Mg: Ag capped with ITO [86].

The Laurand group [87] generated a model for the optimization of laser-based sensors. The authors further explored the relative advantages of using organic semiconductors as a laser material rather than dyes in a matrix. This study additionally outlines the general structure and operative principles of this sensor, and the authors were able to demonstrate that experimental bulk and surface sensing data utilizing oligofluorene truxene macromolecules and a conjugated polymer for the gain region were consistent with model-based predictions, underscoring the utility of this biosensor. Finally, these researchers compared organic semiconductor and dye-doped laser sensitivity, generated theoretical and experimental biosensing data, and provided methods for the improvement of the sensitivity of these biosensors [87].

Biorecognition is a key biological process that is commonly leveraged for healthcare and technological applications. In this study, the Biscarini group was able to develop an electrolyte gated field effect transistor (EGOFET). This device was shown to be highly sensitive and specific, enabling the efficient and quantitative assessment of thermodynamics associated with interactions between a human antibody and its cognate antigen at a solid/liquid interface. At TNFα levels <1 nm, this EGOFET biosensor exhibited a super-exponential response, and it had a 100 pm limit of detection. EGOFET sensitivity was ultimately dependent upon analyte concentrations, with maximal sensitivity being achieved in clinically-relevant TNFα concentration ranges provided the sensor was undergoing sub-threshold regime operations. When TNFα levels were >1 nm, EGOFET biosensor responses were found to scale linearly with analyte concentration. Gate voltage was also found to impact the dynamic range and sensitivity of these biosensors. The mechanism was proposed to explain the correlations between sensitivity and the density of states (DOS). Through their analyses of gate voltage-dependent responses, the authors were able to define the binding constants for these interactions and to assess surface charges and effective capacitance related to biorecognition reactions on the surface of their biosensor. Importantly, the authors were able to use this biosensor to detect TNFα in human plasma, underscoring the potential utility of this sensor in healthcare settings [70].

### 3.2. Integrated Biosensor with Complementary Metal-Oxide-Semiconductor (CMOS) Compatible

Dominguez’s group demonstrated the design, development, and evaluation of nano/microbiosensors which were based on optical waveguides in a highly sensitive interferometric configuration and by using evanescent wave detection [88]. To achieve elevated sensitivity for the designed biosensors, they were first precisely designed. Fabrication of the designed biosensors was then achieved through standard silicon CMOS microelectronics technology. Employing two different technologies, two integrated interferometric devices (Mach–Zehnder interferometrics (MZI)) were developed which include (a) MZI micro-device developed through the Anti Resonant Reflecting Optical Waveguide (ARROW) waveguide, and (b) MZI micro-device developed through TIR waveguide. The developed biosensors were then subjected to testing for their capability of direct biosensing after the coupling of the surface device with specific receptors through the nano-scale immobilization technique. The authors concluded that a laboratory-on-a-chip micro-system can be developed through further integration of the micro-optical sensors, photodetectors, microfluidics, and CMOS electronics.

### 3.3. Metal-Organic Frameworks as Biosensors for Luminescence-Based Detection and Imaging

In this article, the Fairen-Jimenez’s group [89] describes metal-organic frameworks (MOFs) generated through the self-assembly of organic linkers and metal clusters or centers. These MOFs exhibit important chemical and structural properties that make them ideally suited for use in biomedical, chemical, and environmental biosensing applications. Notably, these MOFs are highly porous, exhibit significant crystallinity, have a tunable composition, possess a large surface area, and are amenable to molecular modifications. Through a review of the relevant literature, these authors discuss classes of MOF-based biosensors that have been designed for qualitative sensing (including materials designed for MRI contrast and fluorescence microscopy) and quantitative sensing (including luminescence-based sensors that can be leveraged to directly assess analyte concentrations). Through summaries of the pertinent literature, these authors underscore the promise of the MOF field as a platform for the development of novel biosensors and imaging technologies [89].

## 4. Conclusions and Perspectives

We have surveyed in this article utilizing semiconductor structures for biosensor application by explicitly concentrating on the application as the detecting interface for wide bio identification and therapeutic analysis.

The quantity of production on those points is rapidly expanding mirroring the solid interest in a clinical application. Because of trademark properties, for example, high affectability, strength, and unwavering quality, semiconductors seem to satisfy a solid need. In any case, for further improvement, explicit applications for which these particular applications become novel (not effectively accomplished by other material) must be investigated.

The In_2_O_3_ FET-included wearable biosensors with respect to chip gold side entryway cathodes can be utilized for very touch-sensitive recognition of glucose with a location limit down to 10 nm. In_2_O_3_ nanoribbon exhibits showed here will empower these applications and extend minimal effort, huge region designing methodologies to empower an assortment of materials, and structure geometries in nanoelectronics. Self-assembled graphene FET biosensors beat the coupling partiality subordinate affectability of nucleic acid biosensors and offer a pathway toward multiplexed and mark-free nucleic corrosive testing with high precision and selectivity.

Monolayer MoS_2_ interfaces depicted here yield a wide accumulation of results identified with materials, gadgets, and applications parts of the utilization of MoS2 as separated precious stones and huge zone polycrystalline films in biodegradable electronic frameworks. Membraneless FET biosensors recommend a compelling stage for future touchy and reusable biosensors dependent on deformity-free VDW materials. The upgraded PEC current acquired utilizing the wrinkled materials design empowered us to build up a delicate and mark-free DNA biosensor with a picomolar limit-of-discovery [84].

GFETs defeat the current 2 D sensor shortage upon the ability of biosensors and offer a platform toward multiplexed and marker-free testing with high precision and selectivity. The significance of bio-detection and clinical determination will increasingly be upgraded. Despite the fact that there are solid requests for research of semiconductors’ interesting properties, we will be ready to recognize new applications for these semiconductors.

## Data Availability

Not applicable.
