# Correction: Zhang et al. Recent Progress on Semiconductor-Interface Facing Clinical Biosensing. *Sensors* 2021, *21*, 3467

**DOI:** 10.3390/s26020485

**Published:** 2026-01-12

**Authors:** Mingrui Zhang, Mitchell Adkins, Zhe Wang

**Affiliations:** 1 School of Engineering, University of Manchester, Manchester M13 9PL, UK; mingrui.zhang-2@student.manchester.ac.uk; 2 Chemistry Department, Oakland University, Rochester, MI 48309, USA; mitchelladkins@oakland.edu

**Text Correction**

In the original publication [1], some terms, like “precious stones” and so on, were used in a few instances. As these terms are not commonly used, they have been replaced with more appropriate terms to improve clarity. The corrected content appears below.

1. Introduction

Paragraph 3: “precious stones” is replaced by “crystals”, and “information entryway” is replaced by “input gate”.

2.1.1. Nanofabrication-Affiliated FET

Paragraph 3: “Nuclear power micrographs” is replaced by “Atomic Force Micrographs”, and “Filtering electron microscopy” is replaced by “Scanning Electron Microscopy (SEM)”.

Paragraph 6: “Barrette” is replaced by “Hairpin”, and “clasp” is replaced by “clamp”.

Paragraph 10: “movies” is replaced by “films”.

2.1.2. Membraneless Field-Effect Transistor (FET) Biosensors

Paragraph 3: “non-membrane” is replaced by “membrane-free”.

2.2. Photoelectrochemical Biosensor

Paragraph 2: “movies” is replaced by “films”.

3.1. Organic Semiconductor Interface for Biosensor

Paragraph 3: “movies” is replaced by “films”.

4. Conclusions and Perspectives

Paragraph 3: “nucleic corrosive” is replaced by “nucleic acid”.

Paragraph 4: “movies” is replaced by “films”.

The authors state that the scientific conclusions are unaffected. This correction was approved by the Academic Editor. The original publication has also been updated.
